# The utility of diffusion-weighted imaging for differentiation of phyllodes tumor from fibroadenoma and breast cancer

**DOI:** 10.3389/fonc.2023.938189

**Published:** 2023-03-02

**Authors:** Jinzhi Fang, Yuzhong Zhang, Ruifeng Li, Lanlan Liang, Juan Yu, Ziqi Hu, Lingling Zhou, Renwei Liu

**Affiliations:** ^1^ Department of Radiology, Affiliated Longhua People’s Hospital, Southern Medical University (Longhua People’s Hospital), Shenzhen, China; ^2^ Clinical Medical College of Dali University, Dali, China; ^3^ Department of Radiology, Affiliated Hospital of Yunnan University, Kunming, China; ^4^ Department of Radiology, The First Affiliated Hospital of Shenzhen University, Health Science Center, Shenzhen Second People’s Hospital, Shenzhen, China

**Keywords:** diffusion-weighted image (DWI), apparent diffusion coefficient (ADC), value, magnetic resonance imaging (MRI), breast tumors, phyllodes tumors

## Abstract

**Objective:**

To evaluate the utility of apparent diffusion coefficient (ADC) values for differentiating breast tumors.

**Methods:**

The medical records of 17 patients with phyllodes tumor [PT; circular regions of interest (ROI-cs) n = 171], 74 patients with fibroadenomas (FAs; ROI-cs, n = 94), and 57 patients with breast cancers (BCs; ROI-cs, n = 104) confirmed by surgical pathology were retrospectively reviewed.

**Results:**

There were significant differences between PTs, FAs, and BCs in ADCmean, ADCmax, and ADCmin values. The cutoff ADCmean for differentiating PTs from FAs was 1.435 × 10^−3^ mm^2^/s, PTs from BCs was 1.100 × 10^−3^ mm^2^/s, and FAs from BCs was 0.925 × 10^−3^ mm^2^/s. There were significant differences between benign PTs, borderline PTs, and malignant PTs in ADCmean, ADCmax, and ADCmin values. The cutoff ADCmean for differentiating benign PTs from borderline PTs was 1.215 × 10^−3^ mm^2^/s, and borderline PTs from malignant PTs was 1.665 × 10^−3^ mm^2^/s.

**Conclusion:**

DWI provides quantitative information that can help distinguish breast tumors.

## Introduction

Phyllodes tumor (PT), first introduced by Muller in 1838, is a fibroepithelial neoplasm that is histologically similar to a fibroadenoma (FA). PTs are rare, accounting for 2% to 4.4% of all diagnosed FAs in one institution (1). Breast magnetic resonance imaging (MRI) has an overall sensitivity of 90% and a specificity of 72% for detecting breast lesions (2, 3). From 2004 to 2019, there were only a few reports describing the characteristics of PTs on MRI (1, 4–8). PTs may be detected on MRI and classified according to the American College of Radiology Breast Imaging-Reporting and Data System (BI-RADS). The BI-RADS evaluates the benign and malignant nature of lesions according to morphological characteristics and kinetic curve assessments. PTs are classified as benign, borderline, or malignant based on semi-quantitative histological features (mitotic phase, degree of stromal dysplasia, and margin) (9). PTs that do not have typical malignant signs but sufficient suspicious manifestations should be classified as BI-RADS IV. PTs exhibit different time-signal intensity curve (TIC) types (10, 11). The TICs exhibited by benign PTs may be similar to FAs, while the TICs exhibited by borderline and malignant PTs may be similar to breast cancers (BCs). TIC type does not correlate with the histologic grade of PTs (5). PTs and FAs may have a contrast enhancement pattern suggestive of malignancy in up to one-third of cases, and some potentially benign lesions cannot be differentiated from BCs (4).

Diffusion-weighted imaging (DWI) has become clinically relevant (12, 13). DWI is a non-invasive MRI technique that can measure the diffusion of water molecules across tissues, *in vivo*. The motion of water molecules in tissues depends on tissue cellularity and the integrity of cell membranes. Differences in the motion of water molecules between tissues cause signal attenuation. To date, DWI for breast tumor applications has relied on the mono-exponential model with b-values of 0 and 800 s/mm^2^ (14–16). Other studies adopted b = 0/1000 s/mm^2^ (17, 18). This assumes an exponential decay in signal intensity with the product of the b value and apparent diffusion coefficient (ADC). ADC values reflect the slope of the best fit straight line to the log signal as a function of the b-value (19). When the b-value is >1000 s/mm^2^, signal intensity corresponds to the anatomical and physiological characteristics of breast tissue and, thus, deviates from the single exponential model. In this case, a bi-exponential model is necessary to measure diffusion and microperfusion parameters. ADC values may be determined in three different types of tumor regions of interest (ROIs), including a circular ROI (ROI-c), single-slice ROI (ROI-s), and whole-tumor ROI (ROI-w) (13). ADC values can provide objective and accurate quantitative information (20–24). ADC values are impacted by ROI selection (8). A smaller ROI placed over the most hypointense ADC area may provide better discrimination performance by reflecting the worst pathology within a heterogeneous lesion, but whole tumor measurement may allow better reproducibility (13). The objective of this study was to evaluate the utility of ADC values to differentiate between PTs, FAs, and BCs, and to classify PTs.

## Materials and methods

### Study subjects

The medical records of female patients diagnosed with breast tumors between 1 January 2017 and 5 April 2022 were retrospectively reviewed. This retrospective analysis of breast MRI data was approved by the Institutional Research Ethics Board of our institute (Approval No. 20220509). The requirement for informed consent was waived. Inclusion criteria were: 1) unilateral or bilateral solid breast tumor, 2) no history of surgery, 3) no history of other tumors or systemic diseases, and 4) surgical pathology provided a definitive diagnosis. All patients underwent MRI examination 3–7 days prior to surgery. Patients were divided into three groups based on pathological findings: Group A, PT; Group B, FA; and Group C, BC.

### MRI protocols

Patients were scanned using a 3.0-T (Ingenia, Philips Medical systems, Netherlands) superconducting MRI scanner. DWI sequences were obtained with b-values of 0 and 800 s/mm^2^. DWI parameters: FOV (mm): RL × AP × FH, 340 × 196 × 150; voxel (mm): 3.04 × 1.87 × 3; REC voxel MPS (mm): 1.06 × 1.06 × 1.06; slice thickness (mm): 3; slice gap (mm): 0; matrix (slices): 112 × 105 × 50; REC matrix: 320; NSA: 2; scan percentage (%): 163.2; total scan duration (min): 3:07; SNR: 1.027; TR (ms): 12500; min.TR (ms): 11007; TE (ms): 82; EPI factor: 93; BW in EPI freq.dir (HZ): 2129.8; WFS (pix)/BW (hz): 24.817/17.5; fold-over suppression: oversampling; P (mm): 153; A (mm): 73; stacks: 1; type: parallel; slices: 50; slice gap: 0; slice orientation: transverse; fold-over direction: AP; fat shift direction: P; packages: 1; local torso SAR: <64%; whole body SAR/level: <1.7 W/kg/normal; SED: <0.3 kj/kg; coilpower: 51%; maxB1 + rms: 1.67 uT. ADC maps were processed using the post-processing software (Philips Intellispace Portal). Two radiologists placed an ROI-c (10–300 mm^2^) on a 2D single-slice of each breast tumor. The area of the ROI-cs (mm^2^), ADCmean, ADCmax, ADCmin, and standard deviation (SD) were calculated.

### Statistical analysis

Statistical analysis was conducted using SPSS v28.0.1. Descriptive statistics, including mean and standard deviation, were summarized for each ADC parameter. Normality of ADC values was evaluated with the single-sample Shapiro-Wilk test. Normally distributed data with homogeneity of variance were compared with ANOVA. Non-normally distributed data with heterogeneous variance were compared with the non-parametric Kruskal-Wallis H test. Pairwise comparison was made with the Mann-Whitney *U* test. The area under the curve (AUC) of receiver operating characteristic (ROC) curves was used to assess the diagnostic performance of ADC parameters for breast tumors. p < 0.05 was considered statistically significant.

## Results

**Figure 1:**
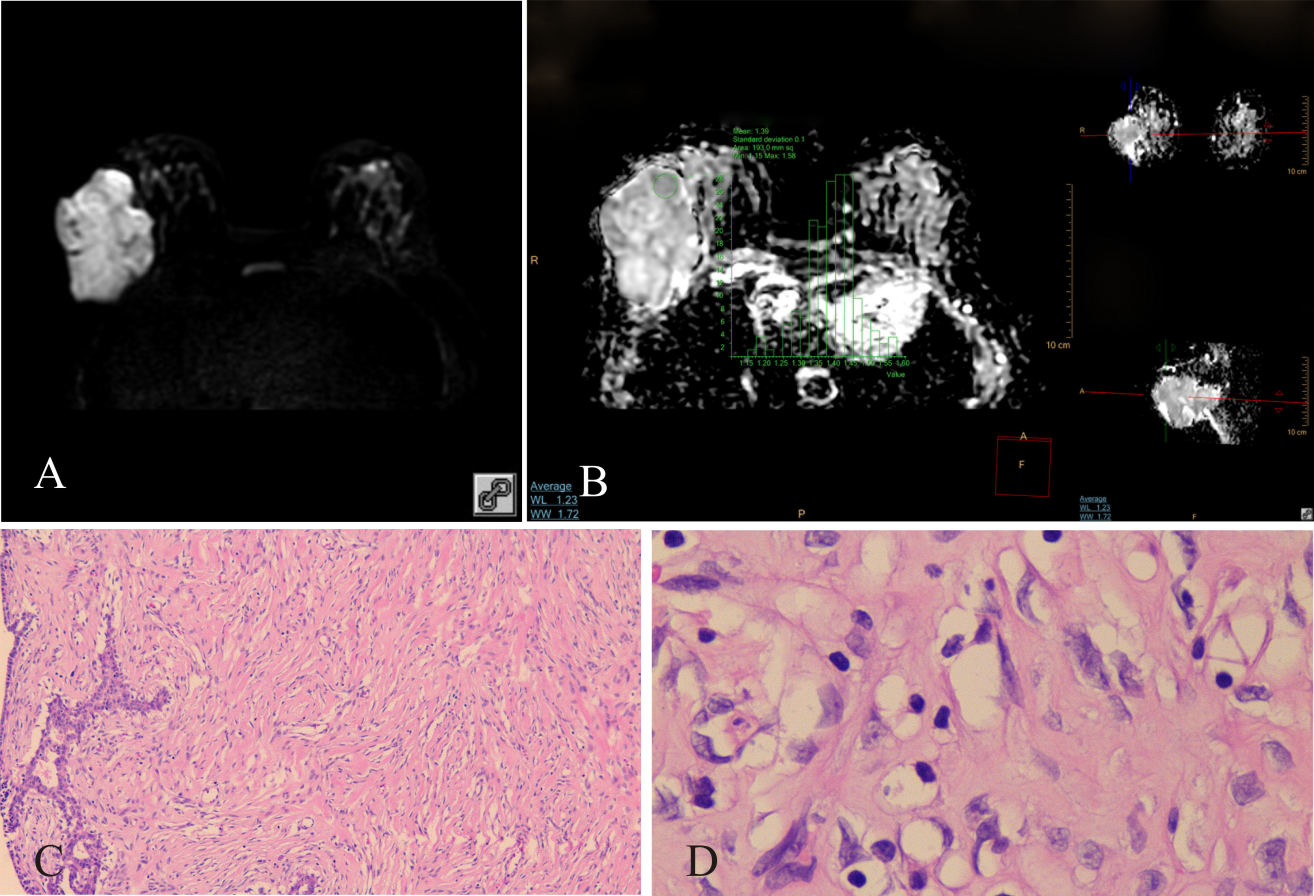
Benign PT of the right breast, female, age 46 years. **(A)** DWI (b800s/mm^2^): mixed hyperintense signal; **(B)** ADC map: isointense–hyperintense mixed signal, ADCmean = 1.39 × 10^−3^ mm^2^/s; **(C)** HE ×100: tumor stromal cells were dispersed; **(D)** HE ×400: no nuclear division was observed, tumor cells were loosely arranged.

**Figure 2:**
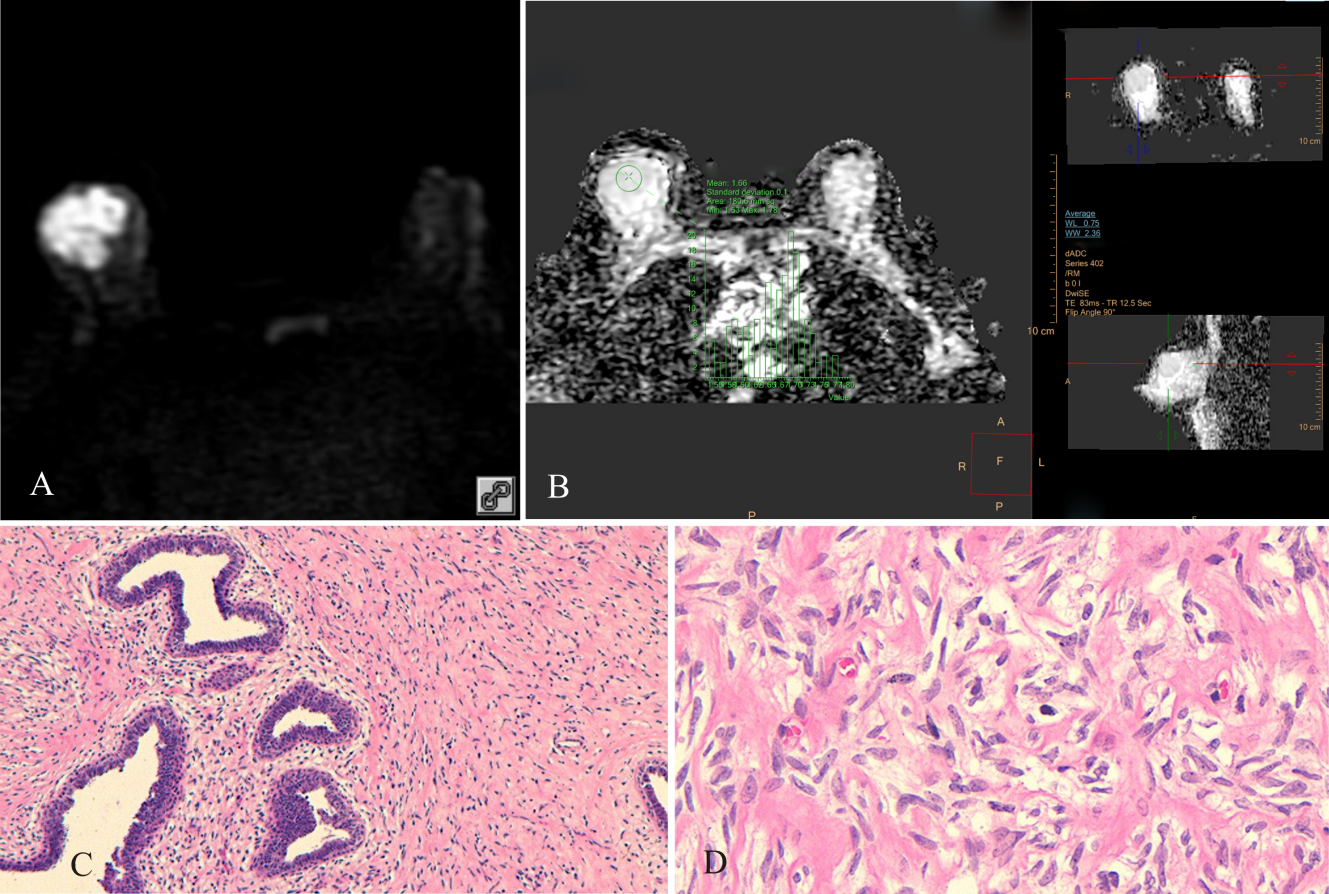
Borderline PT of the right breast, female, age 45 years. **(A)** DWI (b800 s/mm^2^): mixed hyperintense signal; **(B)** ADC map: isointense signal, ADC mean = 1.66×10^−3^ mm^2^/s; **(C)** HE ×100: uneven distribution of tumor stromal cells, high cell density in some areas, low cell density in other areas; **(D)** HE ×400: tumor stromal cells had “tadpole-like” nuclei, cells were closely packed.

**Figure 3:**
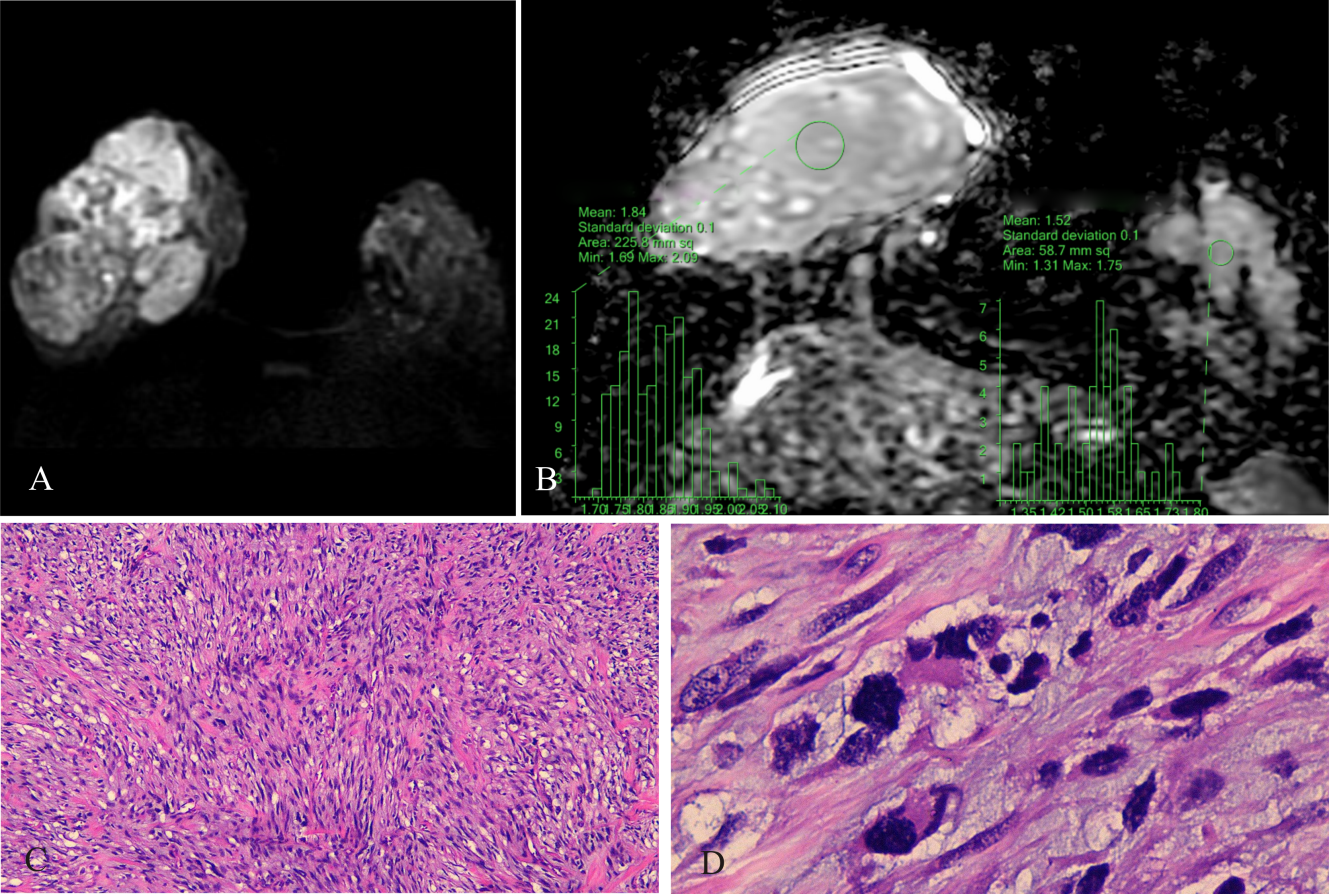
Malignant PT of the right breast, female, age 29 years. **(A)** DWI (b800 s/mm^2^): mixed hyperintense signal; **(B)** ADC map: isointense-hyperintense mixed signal, ADCmean = 1.84 × 10^−3^ mm^2^/s; ADCmean (left normal breast) = 1.52 × 10^−3^ mm^2^/s; **(C)** HE ×100: tumor stromal cells were closely packed; **(D)** HE ×400: nuclear fission, interstitial edema was insignificant.

The medical records of 148 patients with breast tumors were retrospectively reviewed, including 17 patients with PTs [eight benign PTs (**Figure 1**), six borderline PTs (**Figure 2**), and three malignant PTs (**Figure 3**)], 74 patients with FAs, and 57 patients with BCs. A total of 369 ROI-cs were evaluated, including 171 ROI-cs for PTs, 94 ROI-cs for FAs, and 104 ROI-cs for BCs. Patients’ mean (SD) age was 49.17 ± 2.95 years (range, 19–74 years old), and time since diagnosis ranged from 3 weeks to 2 months; 88 patients underwent surgical resection, and 60 patients underwent excisional biopsy.

ADCmean, ADCmax, and ADCmin of PTs were 1.6083 (0.83–2.16) ± 0.26015 × 10^−3^ mm^2^/s, 1.8112 (0.94–2.44) ± 0.28428 × 10^−3^ mm^2^/s, and 1.4113 (0.69–2.05) ± 0.28392 × 10^−3^ mm^2^/s, respectively, which were higher than the ADCmean, ADCmax, and ADCmin of FAs and BCs. Multiple group comparisons conducted with the Kruskal-Wallis H test and Mann-Whitney *U* test showed significant differences *(*p < 0.001) (**Supplementary Tables 1–4**). ROC curve analysis and the Youden index were used to determine optimum cutoff values for ADC parameters for differentiating PTs, FAs, and BCs (**Supplementary Figure 1** and **Table 1**). ADCmean had the largest AUC among ADC mean, ADCmax, and ADCmin. For PTs vs. FAs, the AUC of ADCmean was 0.823 (95% CI 0.764–0.881). For PTs vs. BCs, the AUC of ADCmean was 0.987 (95% CI 0.977–0.996). For FAs vs. BCs, the AUC of ADCmean was 0.906 (95% CI 0.8677–0.946). The cutoff ADCmean for differentiating PTs from FAs was 1.435 × 10^−3^ mm^2^/s, PTs from BCs was 1.100 × 10^−3^ mm^2^/s, and FAs from BCs was 0.925×10^−3^ mm^2^/s.

**Table 1:** Diagnostic performance of ADC parameters for differentiating between PTs, FAs, and BCs.

Parameter	Comparison group	AUC	Cutoff	Sensitivity	Specificity	Youden Index	95% CI	p-Value
ADCmean (×10^−3^ mm^2^/s)	a	0.823	1.435	0.813	0.819	0.632	0.764–0.881	<0.0001
	b	0.987	1.100	0.947	0.942	0.890	0.977–0.996	<0.0001
	c	0.906	0.925	0.947	0.729	0.676	0.867–0.946	<0.0001
ADCmax (×10^−3^ mm^2^/s)	a	0.802	1.575	0.836	0.681	0.517	0.743–0.862	<0.0001
	b	0.952	1.545	0.848	0.962	0.809	0.929–0.976	<0.0001
	c	0.777	1.390	0.585	0.860	0.445	0.714–0.840	<0.0001
ADCmin (×10^−3^ mm^2^/s)	a	0.810	1.245	0.784	0.787	0.571	0.753–0.868	<0.0001
	b	0.986	0.965	0.906	0.971	0.878	0.977–0.996	<0.0001
	c	0.894	0.760	0.915	0.776	0.691	0.850–0.938	<0.0001

a, PT vs. FA; b, PT vs. BC; c, FA vs. BC (AUC of ROI-c and SD was small and were not included in further analyses).

The ADCmeans of benign PTs, borderline PTs, and malignant PTs were 1.5619 (1.25–1.92) ± 0.14886 × 10^−3^ mm^2^/s, 1.3098 (0.83–1.68) ± 0.25017 × 10^−3^ mm^2^/s, and 1.7962 (1.45–2.16) ± 0.13255 × 10^−3^ mm^2^/s, respectively (**Supplementary Table 5**). ADCmean, ADCmax, and ADCmin of benign PTs, borderline PTs, and malignant PTs were significantly different (**Supplementary Tables 6–9**). For benign PTs vs. borderline PTs, the AUC of ADCmean was 0.771 (95% CI 0.672–0.870). For borderline PTs vs. malignant PTs, the AUC of ADCmean was 0.982 (95% CI 0.964–0.999). The cutoff ADCmean for differentiating benign PTs from borderline PTs was 1.215 × 10^−3^ mm^2^/s and borderline PTs from malignant PTs was 1.665 × 10^−3^ mm^2^/s. For benign PTs vs. malignant PTs, the ADCmin had the largest AUC among ADCmean, ADCmax, ADCmin; which was 0.905 (95% CI 0.848–0.961); the cutoff ADCmin was 1.465 × 10^−3^ mm^2^/s (**Supplementary Figure 2** and **Table 2**).

**Table 2:** Diagnostic performance of ADC parameters for classifying PTs.

Parameter	Comparison Group	AUC	Cutoff	Sensitivity	Specificity	Youden Index	95% CI	p-Value
**ADCmean** (×10^−3^ mm^2^/s)	a	0.771	1.215	1.000	0.488	0.488	0.672~0.870	<0.0001
	b	0.879	1.625	0.692	0.949	0.641	0.816~0.943	<0.0001
	c	0.982	1.665	0.976	0.885	0.860	0.964~0.999	<0.0001
**ADCmax** (×10^−3^ mm^2^/s)	a	0.702	1.520	0.923	0.463	0.386	0.595~0.809	0.0009
	b	0.772	1.750	0.577	0.962	0.538	0.684~0.861	<0.0001
	c	0.940	1.785	0.854	0.897	0.751	0.896~0.984	<0.0001
**ADCmin** (×10^−3^ mm^2^/s)	a	0.749	1.080	1.000	0.561	0.561	0.641~0.857	<0.0001
	b	0.905	1.465	0.942	0.833	0.776	0.848~0.961	<0.0001
	c	0.951	1.460	0.951	0.833	0.785	0.916~0.985	<0.0001

a, benign PTs vs. borderline PTs; b, benign PTs vs. malignant PTs; c, borderline PTs vs. malignant PTs.

## Discussion

BI-RADS is a comprehensive guideline used by radiologists for breast tumor classification. Conventional MRI sequences are a complementary approach to assessing breast tumors. DWI technology is not included in the BI-RADS system, but the use of ADC values to evaluate breast tumors has become a research hotspot in recent years (14, 17, 23, 25, 26). The multiparameter MRI model with dynamic contrast-enhanced (DCE)-MRI, DWI, and synthetic MRI is a robust tool for evaluating malignancies in BI-RADS IV lesions. Including clinical features may further improve the diagnostic performance of this model (10). PTs are rare breast tumors that have not been widely recognized by clinicians. Reports on the use of ADC values to analyze PTs are scarce (5, 7, 8). Due to the large size of PTs (7), ROI-cs can be used to obtain ADC values that reflect tumor heterogeneity (14, 15).

Clinically, PTs are usually managed surgically. Benign and borderline PTs require wide excision. Malignant PTs >10 cm or PTs with rapid progression in a short period require whole mastectomy. PTs are likely to recur after surgery, but only malignant PTs develop distant metastases (27). PTs and FAs are difficult to distinguish on breast imaging modalities. On mammography, PTs usually present as rounded, oval, or lobulated masses with well-rounded edges, similar to FAs. On ultrasound, PTs present as well-defined solid, low-echo areas, almost identical to FAs. The sensitivity of fine needle aspiration biopsy for diagnosis of PT is only 40%, and has a high false-negative rate (28). Coarse needle biopsy has a slightly higher sensitivity (approximately 63%) (29), but histopathological examination of the whole tumor is generally required for diagnosis.

In this study, conventional MRI showed that the imaging characteristics of benign, borderline, and malignant PTs overlap, and benign PTs could not be precisely differentiated from other BCs. In previous reports, MRI findings for eight cases of benign PTs identified some characteristics of large benign PTs (>3 cm), but distinguishing small PTs from small FAs was difficult (1); MRI of 24 PTs (n = 1 malignant; n = 23 benign) showed PTs had benign morphological features, administration of contrast material suggested malignancy in 33% of cases, and PTs and FAs could not be precisely differentiated (4); a retrospective review of dynamic MRI findings for 30 cases of PTs (n = 19 benign; n = 6 borderline; n = 5 malignant) showed no significant association between TIC patterns (persistent, plateau, washout) and histopathological findings (5).

According to the results of this study, the ADCmeans of PTs, FAs, and BCs were 1.6083 (0.83–2.16) ± 0.26015 × 10^−3^ mm^2^/s, 1.2711 (0.81–2.20) ± 0.31678 × 10^−3^ mm^2^/s, and 0.8496 (0.60–1.26) ± 0.14857 × 10^−3^mm^2^/s, respectively. The ADCmeans of PTs was significantly higher than those of FAs and BCs *(*p < 0.001). ADCmean had the best efficacy to discriminate between PTs, FAs, and BCs compared to ADCmax and ADCmin, and had the highest specificity. The specificity of ADCmean for differentiating between PTs and FAs or PTs and BCs was 81.90% and 94.2%, respectively. These findings suggest ADCmean has potential as a clinically useful technology. In 2020, Jelena et al. (26) reported that DWI is a clinically useful tool for the differentiation of malignant from benign lesions based on mean ADC values. To the authors’ knowledge, the present study is the first published report comparing the ADC values of PTs, FAs, and BCs.

The ADCmeans of benign PTs, borderline PTs, and malignant PTs were 1.5619 (1.25–1.92) ± 0.14886 × 10^−3^ mm^2^/s, 1.3098 (0.83–1.68) ± 0.25017 × 10^−3^ mm^2^/s, and 1.7962 (1.45–2.16) ± 0.13255 × 10^−3^ mm^2^/s, respectively, and were significantly different. ADC values of malignant PTs at b0/1000 s/mm^2^ have been reported as 1.37 ± 0.03 (10^−3^ mm^2^/s) (5), 1.03 ± 0.03 (10^−3^ mm^2^/s), and 1.45 ± 0.03 (10^−3^ mm^2^/s) (7). DWI is performed using motion-sensitizing gradients applied during MR image acquisition to probe local diffusion characteristics. The resulting diffusion-weighted MRI signal is reduced in intensity proportional to water mobility, and is commonly described by the monoexponential equation: SD=S0 e^−b*ADC^ (13). Theoretically, as the b value increases, the corresponding ADC value should gradually decrease. Therefore, ADC values obtained in this study at b0/800 s/mm^2^ should be greater than those reported at b1000s/mm^2^. This was not always the case, likely due to the heterogeneity of breast tumors (14, 15).

The motion of water molecules in tissues depends on tissue cellularity and the integrity of cell membranes (30, 31). Consequently, PT cellularity should correlate with ADC values. Previous reports show an association between the ADC values of BCs and some histological features (32), and malignant tumors had lower ADC values than benign tumors due to high cellularity in the tumors (33). In the present study, ADC values reflected pathological findings, which showed that malignant and borderline PTs had high cell densities, while tumor cells of benign PTs were more dispersed. However, the ADCmean of malignant PTs was higher than benign or borderline PTs. This may be because the ADCmean of malignant PTs was not only related to tumor cell density, but also to the necrosis, cystic degeneration, and edema occurring inside the tumor. Extensive necrosis and interstitial edema allow water protons to move freely, which strongly influence the ADC value. The cutoff ADCmean has important clinical application. Correct diagnosis of PT grade is required before breast surgery. In our study, PTs were benign at ADCmean > 1.215 × 10^−3^ mm^2^/s or malignant with internal liquefaction, necrosis, and cystic degeneration at ADCmean >1.665×10^−3^ mm^2^/s. ADCmin had clinical application for the differentiation of benign and malignant PTs, and PTs were considered malignant at ADCmin >1.465×10^−3^ mm^2^/s.

## Limitations of the study

This study was associated with several limitations. First, it was a retrospective study, and the clinical value of ADC values for discriminating between breast tumors should be verified in prospective studies. Second, the sample size was small, and there may have been interobserver variability with regard to ROI-c selection, which may have introduced bias. Third, DWI sequences included b-values of 0 and 800 s/mm^2^; further research should include multi-b-value DWI. Fourth, distortion and deformation often occur at high b-values, which may disturb ADC parameters.

## Conclusion

Breast DWI acquiring b0 and 800 s/mm^2^ images took 3 minutes. This enabled us to obtain satisfactory ADC values to evaluate the histological characteristics of a tumor. ADCmean differentiated PTs, FAs, and BCs, and benign PTs from borderline PTs and borderline PTs from malignant PTs. ADCmin helped differentiate benign PTs from malignant PTs. Overall, ADC values provided quantitative information that has potential to distinguish between PTs, FAs, and BCs, and classify PTs.

## Data availability statement

The raw data supporting the conclusions of this article will be made available by the authors, without undue reservation.

## Ethics statement

The studies involving human participants were reviewed and approved by People’s Hospital of Longhua. The patients/participants provided their written informed consent to participate in this study.

## Author contributions

Image evaluation, JF, JY, and RWL; Quality control, YZ and RWL; Literature retrieval, RFL; Data and statistics, LL and RWL; MR scanning, ZH, and LZ; Manuscript writing, RWL. All authors contributed to the article and approved the submitted version.
