# Supplementary material for: The utility of diffusion-weighted imaging for differentiation of phyllodes tumor from fibroadenoma and breast cancer
Source: Front Oncol. 2023 Mar 2;13:938189. doi: 10.3389/fonc.2023.938189 (PMC10018141; doi:10.3389/fonc.2023.938189)
Supplement: Supplementary file 1 [file DataSheet_1.docx]

**
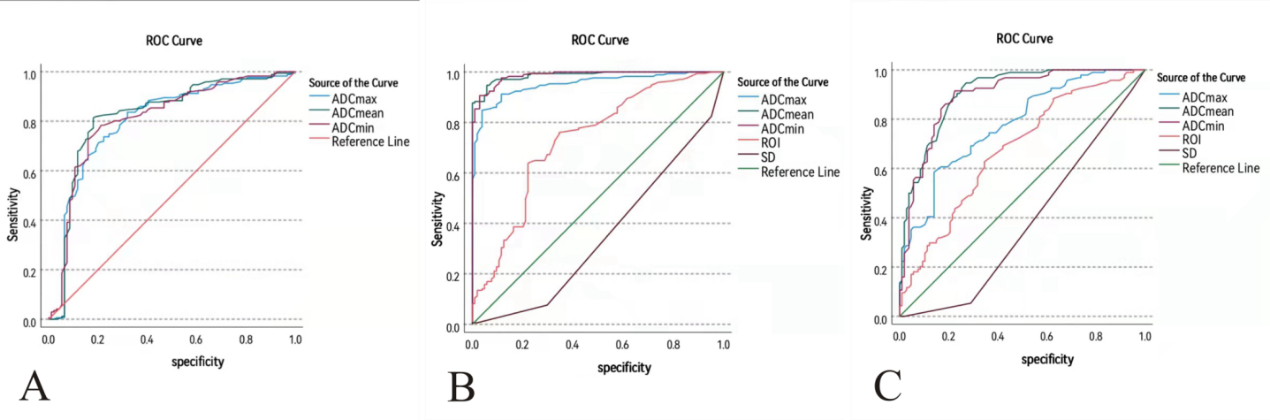
**

**Supplementary Figure 1** Receiver operating characteristic curves **A.** PT vs. FA; **B.** PT vs. BC; **C.** FA vs. BC

**
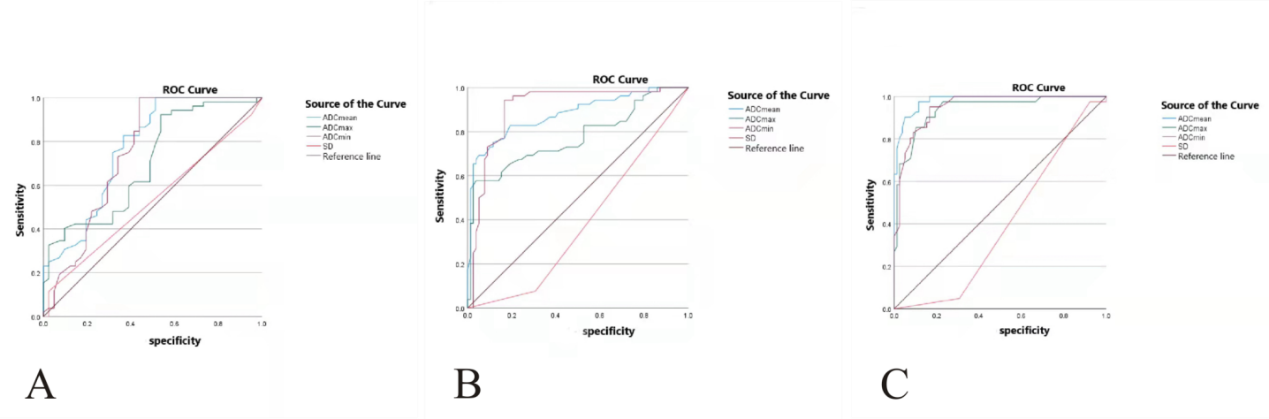
**

**Supplementary Figure 2** Receiver operating characteristic curves **A.** benign PTs vs. borderline PTs; **B.** benign PTs vs. malignant PTs; **C.** borderline PTs vs. malignant PTs
