# Supplementary material for: The utility of diffusion-weighted imaging for differentiation of phyllodes tumor from fibroadenoma and breast cancer
Source: Front Oncol. 2023 Mar 2;13:938189. doi: 10.3389/fonc.2023.938189 (PMC10018141; doi:10.3389/fonc.2023.938189)
Supplement: Supplementary file 2 [file Table_1.docx]

| **Supplementary Table 1 ADC parameters** | | | | | | |
| --- | --- | --- | --- | --- | --- | --- |
| **PARAMETER** | **GROUP** | **N** | **MIN** | **MAX** | **MEAN** | **SD*** |
| ROI-c(mm^2^) | PTs | 171 | 13.50 | 589.30 | 134.0170 | 108.26076 |
|  | FA | 94 | 11.30 | 532.40 | 119.3053 | 103.47789 |
|  | BCs | 104 | 4.50 | 347.70 | 73.9702 | 61.80158 |
| ADCmean(×10^-3^ mm^2^/s) | PTs | 171 | 0.83 | 2.16 | 1.6083 | 0.26015 |
|  | FA | 94 | 0.81 | 2.20 | 1.2711 | 0.31678 |
|  | BCs | 104 | 0.60 | 1.26 | 0.8496 | 0.14857 |
| ADCmax(×10^-3^ mm^2^/s) | PTs | 171 | 0.94 | 2.44 | 1.8112 | 0.28428 |
|  | FA | 94 | 0.97 | 2.56 | 1.4830 | 0.34263 |
|  | BCs | 104 | 0.76 | 1.78 | 1.1728 | 0.20913 |
| ADCmin(×10^-3^ mm^2^/s) | PTs | 171 | 0.69 | 2.05 | 1.4113 | 0.28392 |
|  | FA | 94 | 0.51 | 1.99 | 1.0645 | 0.30895 |
|  | BCs | 104 | 0.08 | 1.21 | 0.6030 | 0.19884 |
| SD | PTs | 171 | 0.00 | 0.60 | 0.0924 | 0.06231 |
|  | FA | 94 | 0.00 | 0.20 | 0.0968 | 0.03725 |
|  | BCs | 104 | 0.00 | 0.30 | 0.1269 | 0.05784 |
|  |  |  |  |  |  |  |

**NOTE:** ROI-c: circular ROI.

**Supplementary Table 2 Normality test**

| parameter | Group | Shaprro-wilk | | |
| --- | --- | --- | --- | --- |
|  |  | **statistics** | **variance** | **P value** |
|  | PTs | 0.771 | 171 | ＜0.001 |
| ROI-c(mm^2^) | FA | 0.774 | 94 | ＜0.001 |
|  | BCs | 0.801 | 104 | ＜0.001 |
|  | PTs | 0.957 | 171 | ＜0.001 |
| ADCmean(×10^-3^ mm^2^/s) | FA | 0.866 | 94 | ＜0.001 |
|  | BCs | 0.952 | 104 | 0.001 |
|  | PTs | 0.978 | 171 | 0.008 |
| ADCmax(×10^-3^ mm^2^/s) | FA | 0.891 | 94 | ＜0.001 |
|  | BCs | 0.981 | 104 | **0.146** |
|  | PTs | 0.979 | 171 | 0.012 |
| ADCmin(×10^-3^ mm^2^/s) | FA | 0.932 | 94 | ＜0.001 |
|  | BCs | 0.977 | 104 | **0.066** |
|  | PTs | 0.570 | 171 | ＜0.001 |
| SD | FA | 0.509 | 94 | ＜0.001 |
|  | BCs | 0.737 | 104 | ＜0.001 |

**NOTE**: ADCmax and ADCmin of BCs 0.146/0.066 ×10^-3^ mm^2^/s, P > 0.05, normal distribution. Non-parametric test was used to compare data.

**Supplementary Table 3 Kruskal-wallisH test**

| PARAMETER | kRUSKAL-WALLISH | VARIANCE | P VALUE |
| --- | --- | --- | --- |
| ROI-c(mm^2^) | 42.206 | 2 | ＜0.001 |
| ADCmean(×10^-3^ mm^2^/s) | 238.136 | 2 | ＜0.001 |
| ADCmax(×10^-3^ mm^2^/s) | 189.197 | 2 | ＜0.001 |
| ADCmin(×10^-3^ mm^2^/s) | 232.882 | 2 | ＜0.001 |
| SD | 32.944 | 2 | ＜0.001 |

**NOTE:** P < 0.05, statistically significant.

| **Supplementary Table 4 Mann-Whitney U test** | | | | | | |
| --- | --- | --- | --- | --- | --- | --- |
|  |  |  |  |  |  |  |
| **PARAMETER** | **COMPARISON**  **GROUP** | **ROI-c** | **ADCmean** | **ADCmax** | **ADCmin** | **SD** |
|  | a | -1.957 | -8.696 | -8.142 | -8.361 | -1.217 |
| Z value | b | -4.115 | -10.317 | -7.022 | -10.068 | -4.188 |
|  | c | -6.381 | -13.531 | -12.571 | -13.527 | -5.251 |
|  |  |  |  |  |  |  |
|  | a | **0.050** | ＜0.0001 | ＜0.0001 | ＜0.0001 | **0.223** |
| P value | b | ＜0.0001 | ＜0.0001 | ＜0.0001 | ＜0.0001 | ＜0.0001 |
|  | c | ＜0.0001 | ＜0.0001 | ＜0.0001 | ＜0.0001 | ＜0.0001 |

**NOTE:** a, PT vs. FA; b, PT vs. BCs; c, FA vs. BCs. There were significant differences between PTs, FAs and BCs in the area of the ROI-cs, ADCmean, ADCmax, ADCmin and SD. Exception: there were no significant differences between PTs and FAs in the area of the ROI-cs and the SD.

| **Supplementary Table 5 ADC parameters** | | | | | | |
| --- | --- | --- | --- | --- | --- | --- |
| **PARAMETER** | **GROUP** | **N** | **MIN** | **MAX** | **MEAN** | **SD*** |
| ROI-c(mm^2^) | Benign PTs | 52 | 23.70 | 512.50 | 169.5346 | 132.57597 |
|  | Borderline PTs | 41 | 23.70 | 462.90 | 128.3439 | 102.17970 |
|  | Malignant PTs | 78 | 13.50 | 589.30 | 113.3205 | 86.64861 |
| ADCmean(×10^-3^ mm^2^/s) | Benign PTs | 52 | 1.25 | 1.92 | 1.5619 | 0.14886 |
|  | Borderline PTs | 41 | 0.83 | 1.68 | 1.3098 | 0.25017 |
|  | Malignant PTs | 78 | 1.45 | 2.16 | 1.7962 | 0.13255 |
| ADCmax(×10^-3^ mm^2^/s) | Benign PTs | 52 | 1.02 | 2.18 | 1.7558 | 0.23789 |
|  | Borderline PTs | 41 | 0.94 | 2.05 | 1.5437 | 0.26420 |
|  | Malignant PTs | 78 | 1.37 | 2.44 | 1.9887 | 0.18046 |
| ADCmin(×10^-3^ mm^2^/s) | Benign PTs | 52 | 1.11 | 1.82 | 1.3344 | 0.12623 |
|  | Borderline PTs | 41 | 0.69 | 1.54 | 1.1088 | 0.24830 |
|  | Malignant PTs | 78 | 0.94 | 2.05 | 1.6217 | 0.19453 |
| SD | Benign PTs | 52 | 0.00 | 0.20 | 0.1038 | 0.04411 |
|  | Borderline PTs | 41 | 0.00 | 0.60 | 0.1073 | 0.08182 |
|  | Malignant PTs | 78 | 0.00 | 0.20 | 0.0769 | 0.05794 |
|  |  |  |  |  |  |  |

**Supplementary Table 6 Normality test**

| parameter | Group | Shaprro-wilk | | |
| --- | --- | --- | --- | --- |
|  |  | **statistics** | **variance** | **P value** |
|  | Benign PTs | 0.861 | 52 | 0.000 |
| ROI-c(mm^2^) | Borderline PTs | 0.759 | 41 | 0.000 |
|  | Malignant PTs | 0.679 | 78 | 0.000 |
|  | Benign PTs | 0.985 | 52 | **0.766** |
| ADCmean(×10^-3^ mm^2^/s) | Borderline PTs | 0.919 | 41 | 0.006 |
|  | Malignant PTs | 0.972 | 78 | **0.084** |
|  | Benign PTs | 0.949 | 52 | 0.028 |
| ADCmax(×10^-3^ mm^2^/s) | Borderline PTs | 0.957 | 41 | **0.128** |
|  | Malignant PTs | 0.972 | 78 | **0.083** |
|  | Benign PTs | 0.936 | 52 | 0.008 |
| ADCmin(×10^-3^ mm^2^/s) | Borderline PTs | 0.927 | 41 | 0.012 |
|  | Malignant PTs | 0.939 | 78 | 0.001 |
|  | Benign PTs | 0.605 | 52 | 0.000 |
| SD | Borderline PTs | 0.240 | 41 | 0.000 |
|  | Malignant PTs | 0.743 | 78 | 0.000 |

**NOT**E: P > 0.05, requires test of homogeneity of variance.

**Supplementary Table 7 Homogeneity of variance test: ADCmean and ADCmax**

| Parameter | ADCmean/ ADCmax | | | |
| --- | --- | --- | --- | --- |
|  | **Based on average** | **Based on median** | **Based on median and adjusted variance** | **Based on post-cut average** |
| Levin statistics | 28.649/6.588 | 26.606/5.107 | 26.606/5.107 | 28.504/6.599 |
| Variance 1 | 2/2 | 2/2 | 2/2 | 2/2 |
| Variance 2 | 168/168 | 168/168 | 165.026/158.843 | 168/168 |
| *P* value | <0.0001/0.002 | <0.0001/0.007 | <0.0001/0.007 | <0.0001/0.002 |

**NOTE:** P < 0.05, non-parametric test was used to compare data.

.

**Supplementary Table 8 Kruskal-wallisH test**

| PARAMETER | kRUSKAL-WALLISH | VARIANCE | P VALUE |
| --- | --- | --- | --- |
| ROI-c(mm^2^) | 3.614 | 2 | **0.164** |
| ADCmean(×10^-3^ mm^2^/s) | 101.532 | 2 | ＜0.001 |
| ADCmax(×10^-3^ mm^2^/s) | 70.273 | 2 | ＜0.001 |
| ADCmin(×10^-3^ mm^2^/s) | 98.098 | 2 | ＜0.001 |
| SD | 11.317 | 2 | 0.003 |

**NOTE:** ROI-c, p>0.05, not statistically significant.

| **Supplementary Table 9 Mann-Whitney U test** | | | | | |
| --- | --- | --- | --- | --- | --- |
| **PARAMETER** | **COMPARISON GROUP** | **ADCmean** | **ADCmax** | **ADCmin** | **SD** |
|  | a | -4.474 | -3.336 | -4.107 | -0.771 |
| Z value | b | -7.314 | -5.253 | -7.803 | -2.848 |
|  | c | -8.617 | -7.875 | -8.060 | -2.337 |
|  |  |  |  |  |  |
|  | a | ＜0.0001 | ＜0.0001 | ＜0.0001 | **0.441** |
| P value | b | ＜0.0001 | ＜0.0001 | ＜0.0001 | 0.004 |
|  | c | ＜0.0001 | ＜0.0001 | ＜0.0001 | 0.019 |

**NOTE:** a, benign PTs vs. borderline PTs; b, benign PTs vs. malignant PTs; c, borderline PTs vs. malignant PTs. There were significant differences between benign PTs, borderline PTs and malignant PTs in ADCmean, ADCmax, ADCmin and the SD. Exception: there was no significant difference between benign PTs and borderline PTs in SD.

.
